# Examining sport tourism role in fostering social sustainability: Qatar youth perceptions

**DOI:** 10.3389/fspor.2024.1388123

**Published:** 2024-05-31

**Authors:** Wadih Ishac

**Affiliations:** Physical Education Department, Qatar University, Doha, Qatar

**Keywords:** social sustainability, resident perception, sport tourism, major sport events, strategic planning

## Abstract

This study examines the evolving perceptions of youth residents in Qatar, particularly university students, regarding the social impact of hosting major sport events from 2015 to 2022. It offers crucial insights into the contribution of sport tourism in fostering social sustainability, contrasting post-event perceptions of the 2015 Handball World Championship (HWC 2015), the 2019 IAAF World Athletics Championship (IAAF 2019), and the 2022 FIFA World Cup (FIFA 2022), using a cross sectional analysis. The impact is assessed across five dimensions: community pride, enhanced community attachment, event excitement, pride in community infrastructure, and community excitement. Participants are categorized based on nationality (Qatari nationals, Arabs excluding Qataris, and Non-Arabs) to capture cultural and demographic differences. Results reveal increasingly positive perceptions across events regarding the five dimensions. Significant multivariate effects are found on the combined dependent variables for event type and nationality. The study underscores the role of hosting sporting events in advancing social sustainability and community development in Qatar. Variations among national groups indicate a need to tailor policies and strategies to foster inclusivity. The work provides valuable baseline insights to inform future hosting bids and encourage regional collaboration.

## Introduction

Sport has become a crucial tourism component, expanding rapidly within the travel industry, influenced significantly by the inherent connection between sport and travel. Widely recognized as one of the fastest-growing sectors in the tourism landscape, this dynamic synergy between sport and tourism has been instrumental in advancing Sustainable Development Goals (SDGs), aligning with the UN 2030 Agenda for Sustainable Development (1). The advancement is achieved by fulfilling the principles of sustainable development, primarily by addressing the needs of the sport community, fostering increased public engagement, and laying the groundwork for heightened public access to sport in the future (2).

The enduring connection between sport and tourism has roots in a lengthy history, with a consensus among most researchers that sporting events constitute a pivotal aspect of event tourism (3) due to the extent of their contribution to tourism growth and expansion (4). In recent times, sustainability has become increasingly prominent across all industries, with a particular focus on sustainable development encompassing economic, social, and environmental outcomes (3). To this end, sporting events play a pivotal role in advancing sustainability by serving as a catalyst for social cohesion. They contribute to cultivating a sense of pride within host communities and nations in general (5). Consequently, assessing citizens’ perceptions of sport events becomes instrumental in shaping policies to enhance social cohesion and community development.

Within this framework, researchers have started to understand and measure the impacts of hosting sport events on residents. While some researchers have focused on the intangible benefits, specifically the social impact of hosting sport events (6–8), others have directed their focus towards the perceived impact associated with such events (9, 10). To evaluate public perception, researchers have examined residents’ intentions to support the relevant event (3, 11, 12), investigated the event's impact on various demographic groups (13), and considered the influence that global pandemics or public health crisis like the COVID-19 have on residents perception before the event (14), during the event, and after the event (15).

To ensure the long-term success of sporting events, Chersulich Tomino et al. (3) emphasize the need for the critical involvement of residents in the comprehensive planning and implementation of the event. Tourism aims fundamentally to enhance residents’ lives and well-being in their communities, relying heavily on the local population's support for sustainable development in tourist destinations (16, 17). Various factors influence residents’ positive attitudes, including economic and socio-cultural elements. For example, Rasoolimanesh et al. (18) discovered that residents’ support for tourism was closely linked to the event's ability to preserve and strengthen socio-cultural norms and values, thereby contributing to social sustainability. Similarly, McKenzie (19) aptly characterizes social sustainability as a “life-enhancing condition within communities, and a process within communities that can achieve that condition” (p.12).

In the Arab World, Qatar stands as a prominent example of sport tourism, mirroring the aspirations shared by many Gulf Cooperation Council (GCC) countries. These nations strategically leverage major sporting events as vehicles to attract a growing influx of tourists. Notably, Qatar achieved a historic milestone by successfully hosting the 2022 FIFA World Cup (FIFA 2022), becoming the first Arab nation to do so. This achievement underlines Qatar's commitment to positioning itself as an international and regional sports hub, while also aiming to become a high-quality tourist destination (20). Such efforts are in line with Qatar National Vision 2030 (QNV 2030), which offers a framework for implementing national and international development strategies. For instance, the new strategy outlined by the Qatar Olympic Committee (QOC) (2023–2030) centers on continuous encouragement and support for various segments of society to engage in sports and adopt a continuous healthy lifestyle (21). Additionally, the strategy prioritizes the ongoing hosting of major sporting tournaments to boost tourism, enhance the national economy, and further invest in the sporting legacy accumulated from previous major sport tournaments held in the country.

Following the above, it becomes crucial to understand the social impact of hosting sports events in Qatar. This understanding provides valuable insights into how they affect local communities, cultures, and social dynamics. Additionally, it aids in assessing the extent to which these events contribute to achieving national development goals. Moreover, this examination provides an opportunity to reassess policies and initiatives aimed at promoting sports and healthy lifestyles for all, thereby aligning with the strategic objectives outlined in the QOC's strategy (2023–2030).

In Qatar, there have been limited studies to examine the impact of hosting major sporting events on residents. Ishac et al. (22) and Ishac and Swart (13) investigated the impact on youth residents in Qatar following various international sporting events, such as the Handball World Championship (HWC 2015) and the IAAF 2019 World Athletic (IAAF 2019) Championship. Additionally, Al-Emadi et al. (23, 24) and Ishac et al. (25) investigated the pre-event perception of Qatar residents towards FIFA 2022.

While previous studies investigated residents’ perceptions towards FIFA 2022 before its occurrence, this research delves into post-event perceptions, highlighting how major sporting events can be a catalyst for advancing SDGs. There is an absence of follow-up research to track the evolution of these perceptions over time. This study aims to identify both similarities and differences in residents’ perceptions and the overall social impact of each event. By investigating how these events affect local communities, culture, and social dynamics, the study sheds light on their role in fostering social cohesion and social inclusion. This comparative helps evaluate the relative effectiveness of different events in generating lasting positive contributions to society. Notably, this research is pivotal in determining whether these events continue to provide enduring positive contributions to society. Through the evaluation and comparison of events impacts, there is potential to enhance their overall quality and leverage them to foster social sustainability and boost sports tourism.

Qatar's commitment to youth development is evident in initiatives encouraging engagement in sports for a healthier lifestyle, as articulated by the Qatar Olympic Committee (26). In addition, the General Secretariat for Development Planning underscores the value of youth in achieving QNV 2030, mainly through increased participation in sports, aligning with the study's focus (27). Given their role as custodians of the next generation and their importance in shaping national development, youth were selected as the target population (13). This focus holds particular significance for Qatar, where 82.44% of the population falls between the ages of 15 and 54 (28). Therefore, by highlighting the role of youth in shaping national development and sustainability goals, the study underscores the importance of considering youth perspectives in policy-making and community development initiatives.

For the purpose of comparing the outcomes of the three events, this study categorizes participants based on their nationalities. As noted by Ishac and Swart (13), Qatari nationals constitute only 11.65% of the population, with the majority of residents in Qatar being non-Qatari citizens (29). Hence, it becomes imperative to delve deeper and analyze the impact of these events while considering different demographic variables. For instance, Chun et al. (30) and Wicker and Sotiriadou (31) have highlighted that specific demographic factors such as gender, age, and nationality may influence the expression of higher levels of psychic income. As a result, by categorizing participants based on nationality, the study extends the theoretical understanding of how different demographic groups perceive and experience the different types of major sporting events. This nuanced analysis contributes to insights into the complex dynamics of event participation and outcomes, informing strategies for enhancing inclusivity and diversity in event planning and management.

The primary objective of this study is to build on the approach proposed by Ishac et al. (22) and Ishac and Swart (13), by conducting a comparative cross-sectional study that compares the post-event perceptions of youth residents’, particularly university students, to the impact of the HWC 2015, the IAAF 2019, and the FIFA 2022, taking nationality of participants into consideration. Emphasizing the significance of youth as future leaders and vulnerable populations, this study underscores the importance of their perspectives in shaping long-term policies for community sustainability.

The study’s findings shed light on the impact of hosting major sport events on youth across different nationalities, serving as a valuable resource for scholars and local sports event organizers. These insights are crucial for informing decision-makers at local and regional levels, providing a comprehensive understanding of the effect of major sporting events on communities. Drawing from Qatar's experiences, the analysis serves as a model to illustrate how hosting such events can foster holistic community development. This model can serve as a guide for neighboring countries considering similar endeavors, showcasing the potential for leveraging event opportunities for social sustainability, community development, and responsible practices.

### Literature review

#### Event sustainability

Sustainability is generally defined as the ability to maintain new ways of working at a certain pace or level, often transforming existing systems to support change (3, 32). When assessing sustainability, many researchers employ a triple-bottom-line framework encompassing economic, environmental, and social dimensions (32–35). Generally, scholars adopting the triple bottom line approach emphasize that economic and environmental legacies, such as new infrastructure and enhanced environmental policies, are overemphasized in sustainable legacy analysis as they are less complex to evaluate and quantify compared to social legacies (36). Other scholars have evaluated the sustainability of major events through the framework of stakeholder theory, assessing various stakeholder interests and community participation in sustainable tourism development (18). Cavagnaro and Curiel (37) proposed a more holistic approach to understanding sustainable event management, arguing for the need to account for sustainable development at the societal, organizational, and individual levels.

Increasingly, scholars have narrowed their analysis to assess the social sustainability dimension of major events, including the intangible elements such as the development of new business networks, civic pride, and destination image (34, 38). For instance, researchers have emphasized the social impact of events on the local communities of host countries, including applying the frameworks of Social Exchange Theory (SET), social capital, and psychic income to understand how residents perceive the hosting of major events (13, 32, 34, 39). In a recent study of social sustainability in the context of local community events, Stevenson (39) observed that “community events encompass small-scale processes and practices that…enact some aspects of social sustainability” (p. 3). While community events often disrupt regular routines and require planning and organization, they benefit the residents in return (39). These events contribute to increased social capital, heightened community participation, positive attachment to place, and enhanced well-being (39). Furthermore, hosting these events plays an essential role in achieving event sustainability, as they can be considered a critical driver for social cohesion (3) and fostering a sense of pride for the host communities and the country in general (5, 8, 40).

Nonetheless, the impacts of events, often referred to as legacies, depend on various factors, such as the size of the event; researchers agree that larger events tend to generate more noticeable impacts, both positive and negative (3). Hosting sport events not only serves as a platform to showcase the culture of a country but also contributes to fostering a sense of community, establishing positive connections with community pride and national identity, and increasing interaction between visitors and residents (8, 11, 23). However, hosting communities may be adversely affected by events due to issues such as increased crime, cultural conflicts, congestion, pollution, and increased cost of living (41, 42). Therefore, assessing citizens’ perceptions of sports events can help formulate social cohesion and community development policies.

#### Residents’ perceptions of psychic income

Within the general framework of sustainability, the social impact is conceptualized in terms of both substantive (ends) and procedural (means) dimensions (43). Mair et al. (34) categorize the social impacts of major events into direct impacts on residents, encompassing factors such as increased volunteering, education, social cohesion, civic pride, and inclusion. Hence, evaluating residents’ perceptions is essential for understanding the generated impact comprehensively and determining strategic directions for future initiatives.

In general, studies investigating perceived social impacts often draw from SET. This is primarily because SET places significant emphasis on residents’ perceptions regarding awareness, attitude, and intention (11, 44, 45). For instance, Ritchie (46) expressed that within the local community, a sense of feeling of pride and enthusiasm can be associated with the international recognition associated with hosting international sport mega-events. This type of sentiment has been described as the “feel-good factor” or psychic income (47–49). Derived from SET, psychic income emerges as a contrasting concept commonly utilized in the context of sports. Crompton (50), a prominent scholar in this field, has utilized the concept of psychic income to evaluate the effects of sporting events. He presented a thorough framework for evaluating psychic income within communities, comprising of seven components: (a) community pride resulting from increased visibility, (b) civic pride from being a sports event host city, (c) pride in efforts to resuscitate deteriorated areas, (d) enhanced collective self-esteem, (e) tangible focus for social bonding, (f) excitement from the event visitors, and (g) emotional involvement with a sport event.

Numerous studies have delved into the psychic income generated from hosting sporting events, utilizing Crompton's methodology. Among these, Kim and Walker (51) stand out for applying five dimensions derived from Crompton’s framework. Their study assessed community pride/image, community attachment, event excitement, community excitement, and community infrastructure (a framework also adopted in this study). Similarly, Kim et al. (40) examined the psychic impact experienced by residents of the Korean Grand Prix using a three-factor model that accounted for both positive and negative psychological effects, including perceived economic benefits, community pride, and community development. Following the same theory, Ishac et al. (25) examined residents’ perceptions before the 2022 FIFA World Cup and found a significant correlation between their perceptions, community attachment, pride, and excitement for the event. A recent study by Mourão et al. (52) assessed the psychic income perceived by local residents at the Rio Olympic Games in 2016, where they found that residents’ psychic income increased when comparing results from pre- to post-event. Furthermore, Wallstam and Kronenberg (53), after assessing the role of major sport events in regional communities, found that the assessed group perceived a stronger positive emotional impact among different international sport events that took place in the same year.

It is essential to understand the shifts in the various dimensions of psychic income and its perception among youth residents, especially university students, regarding the impact of HWC 2015, IAAF 2019, and FIFA 2022. This comparative analysis aids in understanding the effectiveness of event strategies and initiatives and ultimately informs future decision-making processes related to event planning, community engagement, and social sustainability initiatives.

#### Research context

Over the last decade, significant investments have been made in the sports industry across the Arab world. Like its GCC neighbors, Qatar had its sights set on something big. The country was working hard to design strategies to host major sport events with the aim of attracting a considerable influx of tourists (20). Hosting these sporting events are crucial in advancing sustainability, given their role as a catalyst for social cohesion (3) and cultivating a feeling of pride among host communities and nations at large (5). Consequently, evaluating citizens’ views on sports events has become integral in shaping policies to promote social cohesion and advance community development; assessing and comparing sport events impacts over a period of time will provide valuable insight for decision-makers. This prompts the following question: Has hosting several major sporting events generated satisfaction amongst university students residing in Qatar? Has the effectiveness of different events resulted in lasting positive contributions to society? Has hosting these events contributed to fostering social sustainability?

Derived from the notion of social impact, there have only been a handful of studies in Qatar that have examined the impact of hosting major sporting events on residents beyond the FIFA World Cup. Ishac et al. (22) and Ishac and Swart (13) investigated the impact on youth residents in Qatar following various international sporting events, such as the 2015 HWC and the 2019 IAAF World Athletics Championships. They utilized a scale developed by Kim and Walker (51) based on Crompton's (50) concept of psychic income. Ishac et al. (22) adapted the questionnaire to fit the context of the 2015 HWC, finding a positive impact on youth in Qatar, and supporting the QNV 2030. Similarly, Ishac and Swart (13) modified the questionnaire for the 2019 IAAF World Athletics Championships, categorizing residents based on nationality and gender. They proceeded to measure and compare the psychic income across various groups. Their study forecasted the psychic income for the 2022 FIFA World Cup based on the outcomes of the IAAF 2019 event. Through the application of structural equation modeling, they evaluated how both gender and nationality influenced the perceptions of participants. The results indicated that Qatari youth perceived a higher impact compared to their Arab and non-Arab counterparts, with females expressing a higher level of impact than males.

Following the 2022 FIFA World Cup, the QOC unveiled its strategy (2023–2030), which emphasizes the consistent encouragement and support for diverse segments of society to actively participate in sports and regularly embrace a healthy lifestyle. Moreover, the strategy places a significant focus on continuing to host major sporting tournaments to boost tourism, improve the national economy, and build upon the sporting legacy established by previous major events held in the country (21).

Building upon these research findings and in conjunction with the updated goals of the QOC strategy, this study aims to investigate the post-event perceptions of youth residents, particularly university students, regarding the impact of major sporting events (HWC 2015, IAAF 2019, and FIFA 2022) in Qatar. By conducting a comparative cross-sectional analysis, the research aims to identify similarities and differences in residents’ perceptions and assess the overall effectiveness of these events in generating lasting positive contributions to society.

As a result, the study proposes the following hypotheses:

**Hypothesis 1:** The post-event perceptions of youth residents regarding the impact of major sporting events (HWC 2015, IAAF 2019, and FIFA 2022) will exhibit notable variations, indicating different levels of effectiveness in generating lasting positive contributions to society.
**Hypothesis 2:** Youth residents’ perceptions of the impact of major sporting events (HWC 2015, IAAF 2019, and FIFA 2022) will vary based on factors such as event type, nationality, reflecting the diverse socio-cultural dynamics influencing their perspectives.

## Materials and methods

### Item generation

Following the approach proposed by Ishac et al. (22) and Ishac and Swart (13), this study is to investigate the post-event perceptions of youth residents, regarding the impact of major sporting events (HWC 2015, IAAF 2019, and FIFA 2022) in Qatar. The sample primarily targets university students, being the generation that will reshape the future of the country, as previously highlighted. Given its status as the largest university in the country, the focus was on Qatar University (QU) students. According to QU's Fact Book (2023), the student body comprises of 17,172 Qataris (68.7%) and 7,816 non-Qataris. Additionally, there are 18,711 female students (75%) and 6277 male students (54). This proportionate representation aligns with the approach used for the 2019 IAAF participants (13). Results reflecting a similar proportion will be deemed representative (55).

The study utilized a structured survey instrument consisting of 18 questions distributed across five dimensions: community pride (CP), enhanced community attachment (EC), event excitement (EE), pride community infrastructure (PI), and community engagement (CE). The survey instrument was adapted from previous studies (13, 22, 40, 50, 51) and assessed participants’ attitudes using a seven-point Likert scale, ranging from 1 (strongly disagree) to 7 (strongly agree). To ensure relevance to the specific context of each event assessed, slight modifications were made to the survey instrument during data collection.

### Data collection

For each of the three events studied, the author (fluent in both English and Arabic) prepared the questionnaire. The questionnaire was then submitted to the Institutional Review Board for approval. Once approved, the questionnaire was distributed about a month after each event ended. An email was then sent to Qatar University (QU) students explaining the purpose of the study and providing a link to an online survey. The survey included a consent form for participants to agree to before taking part. Around a month after the survey was initially sent out, any incomplete responses were removed from the dataset. Finally, the collected responses were analyzed.

Across the three events, the total number of participants was 764, with 232 from the HWC 2015, 316 from the IAAF 2019, and 216 from the FIFA 2022. The distribution of participants was 25.39% male and 74.61% female. Regarding nationalities, the majority were Qatari (59.95%), followed by Arab (27.75%) and Non-Arab nationals (12.3%). Each dataset adhered to consistent methodologies outlined in prior publications by Ishac et al. (22) for the Handball World Championship and Ishac and Swart (13) for the IAAF World Championship. Although the 2022 FIFA World Cup data methodology was new, intentional design ensured comparability, enabling reliable aggregation and analysis across events.

### Data analysis

This study employs a comparative cross-sectional approach, examining data collected during three different sports events at distinct periods. The design enables the investigation of variations and potential trends in participants’ perceptions over these periods. The initial step involved calculating descriptive statistics to determine means and standard deviations for each item and dimension across all events. Subsequently, a multivariate analysis of variance (MANOVA) was conducted to assess the combined effects of independent variables on dependent variables. To delve deeper, individual analysis of variance (ANOVA) for each dependent variable was performed, providing more specific insights into the effects of each independent variable (56).

Furthermore, to explore differences between individual group means and gain a more nuanced understanding of significant interaction effects, post-hoc comparisons were conducted using the Bonferroni method (57). It is important to highlight that the primary independent variables, including HWC 2015, IAAF 2019, and FIFA 2022, were categorized with the “Event” variable serving as a temporal marker to capture any changes over time, while the “Nationality” variable was used to indicate the three different categories (Qatari, Arabs, and Non-Arabs) capturing cultural and demographic differences among participants. Respectively, the Dependent variables were the 5 dimensions mentioned earlier in Ishac and Swart (13).

## Results

Data collected assessed community perceptions of the impact of hosting major sporting events in Qatar. The survey was conducted across three different samples corresponding to major international sports events: the Handball World Cup in 2015, the IAAF World Championships in 2019, and the FIFA World Cup in 2022. Table 1 presents the results from the different dimensions of CP, EC, EE, PI, and CE along the three events.

**Table 1:** Qatar youth's perceptions on sport event hosting: measurement model results.

Code	Items/dimensions	HWC 2015		IAAF 2019		FIFA 2022	
		Mean	SD	Mean	SD	Mean	SD
CP1	Qatar gained a positive image as the host country	6.11	1.39	6.15	1.30	6.73	0.93
CP2	Qatar showed the ability to host a major sport event	6.26	1.36	6.35	1.22	6.79	0.86
CP3	The hosted event gave opportunities to showcase the country	6.19	1.32	6.34	1.10	6.69	1.00
CP4	The host allowed foreigners to know more about Qatar	5.98	1.41	6.26	1.17	6.61	1.02
***CP***	***Community Pride***	***6***.***15***	***1***.***21***	***6***.***28***	***1***.***01***	***6***.***70***	***0***.***83***
EC1	Hosting the event made residents appreciate their way of life more	4.58	1.80	5.21	1.78	5.97	1.43
EC2	Watching the event increased cooperation among groups in my community	5.20	1.77	5.27	1.75	5.86	1.51
EC3	Watching the event increased my community’s confidence	5.14	1.80	5.66	1.53	5.87	1.60
***EC***	***Enhanced Community Attachment***	***5***.***00***	***1***.***55***	***5***.***38***	***1***.***48***	***5***.***90***	***1***.***31***
EE1	Hosting the event increased my interest in sports	4.37	2.02	5.34	1.78	5.23	2.10
EE2	The event increased my fan involvement	4.27	2.08	5.01	1.95	5.83	1.71
EE3	I enjoyed watching more games	4.67	2.08	4.86	2.08	6.58	1.17
EE4	During the tournaments, the nightlife was more exciting	4.67	2.09	5.29	1.80	6.49	1.19
EE5	During the event, I enjoyed interacting with visitors	4.37	2.02	5.12	1.88	6.25	1.42
***EE***	***Event Excitement***	***4***.***47***	***1***.***80***	***5***.***12***	***1***.***66***	***6***.***08***	***1***.***16***
PI1	Hosting the event improved the quality of city public services	5.33	1.71	5.56	1.52	6.38	1.21
PI2	Hosting the event improved our public and sport facilities	5.81	1.54	6.03	1.22	6.51	1.03
PI3	Presenting the International Sport competition helped urban regeneration	5.32	1.72	5.49	1.55	6.44	1.06
***PI***	***Pride Community Infrastructure***	***5***.***51***	***1***.***50***	***5***.***62***	***1***.***04***	***6***.***44***	***0***.***93***
CE1	The event tournament provided entertainment to the community	5.51	1.63	5.85	1.34	6.55	1.05
CE2	The event tournament brought excitement to the community	5.46	1.70	5.68	1.45	6.70	0.84
CE3	The event provided new activities to the community	5.48	1.65	5.87	1.31	6.45	1.09
***CE***	***Community Excitement***	***5***.***51***	***1***.***57***	***5***.***85***	***1***.***08***	***6***.***57***	***0***.***85***
*** ***	***Overall result***	***5***.***33***	***1***.***31***	***5***.***65***	***1***.***04***	***6***.***34***	***0***.***85***

Bold and Italic refer to the overall result of each dimensions.

The results show, on average, the perceptions of the community's pride, attachment, excitement, and perceived impact increased from the HWC 2015 to the FIFA 2022. Notably, the CP showed an upward trend, with a mean score of 6.15 (SD = 1.21) in 2015, rising to 6.70 (SD = 0.83) after FIFA 2022, which indicates a growing sense of national pride associated with hosting these events. Similarly, EC increased over the three periods, indicating that hosting the events may have contributed to a stronger sense of community among residents. Additionally, EE scores remained relatively high, reflecting consistent enthusiasm and engagement with the events. Together, these trends underscore a sustained excitement and active involvement among residents in response to hosting these major sport events.

PI saw a considerable rise in youth approval, especially concerning the FIFA 2022, indicating significant support for the infrastructure developments associated with it. Likewise, CE followed a similar upward trend, suggesting that entertainment and new activities related to the events were well-received. Overall, there was an observed rise in the overall result throughout these events, with the mean increasing from 5.33 (SD = 1.31) in 2015 to 5.65 (SD = −1.04) in 2019 to 6.34 (SD = 0.85) in 2022.

To better understand the significance of these results, Multivariate Analysis of variance (MANOVA) was conducted. MANOVA demonstrated a significant effect of the “Event”, Wilks’ Lambda = .858, F (10, 1502) = 11.953, *p* < .001, indicating that the type of sporting event had a statistically significant impact on the collective dependent variables. Additionally, “Nationality” exhibited a significant multivariate effect, Wilks’ Lambda = .921, F (10, 1,502) = 6.271, *p* < .001, implying that respondents from different nationalities reported different experiences from these events see Table 2.

**Table 2:** Multivariate tests of effects on variables by event and nationality.

Effect	Wilks’ Lambda	F	Hypothesis df	Error df	*p*-value
Intercept	0.036	4,037.15	5	751	0.000
Event	0.858	11.953	10	1,502	0.000
Nationality	0.921	6.271	10	1,502	0.000
Event * Nationality	0.949	1.97	20	2,491.735	0.006

Similarly, the interaction between “Event” and “Nationality” was also significant, Wilks’ Lambda = .949, F (20, 2,491.735) = 1.970, *p* = .006. The significance of this interaction effect suggests that the impact of the sporting events on the community was not uniform across different nationalities, indicating that some nationalities perceptions varied between the events. These results indicate that both the event's nature and individuals’ cultural background are important determinants of the social impact of hosting international sporting events. Understanding these varied experiences becomes crucial for fostering inclusive and sustainable community development in light of the growing emphasis on social sustainability.

Following the Multivariate Analysis of Variance (MANOVA) results, which highlighted the significant effects of both “Event” and “Nationality” on community perceptions, further analysis through Analysis of Variance (ANOVA) was conducted to explore the impacts of these factors on specific dimensions of community perceptions. For CP, the corrected model was significant [F (7, 755) = 7.368, *p* < .001]. The main effect of the “Event” [F (1, 755) = 13.642, *p* < .001] and the effect of “Nationality” on CP [F (1, 755) = 9.208, *p* = 0.003] were both significant, indicting differences in CP based on specific events and respondents’ nationality (see Table 3).

**Table 3:** Analysis of variance for CP by event and nationality.

Source	Type III sum of squares	df	Mean square	F	*p*-value
Corrected Model	61.413	7	7.767	7.368	<.001
Intercept	18,996.801	1	18,233.853	18,382.000	<.001
Event	27.084	1	13.542	13.642	<.001
Nationality	10.400	1	9.298	9.208	0.003
Event * Nationality	2.955	1	0.739	0.709	0.586
Error	786.594	755	1.042		
Total	31,747.813	764			
Corrected total	848.007	763			

**Figure 1:**
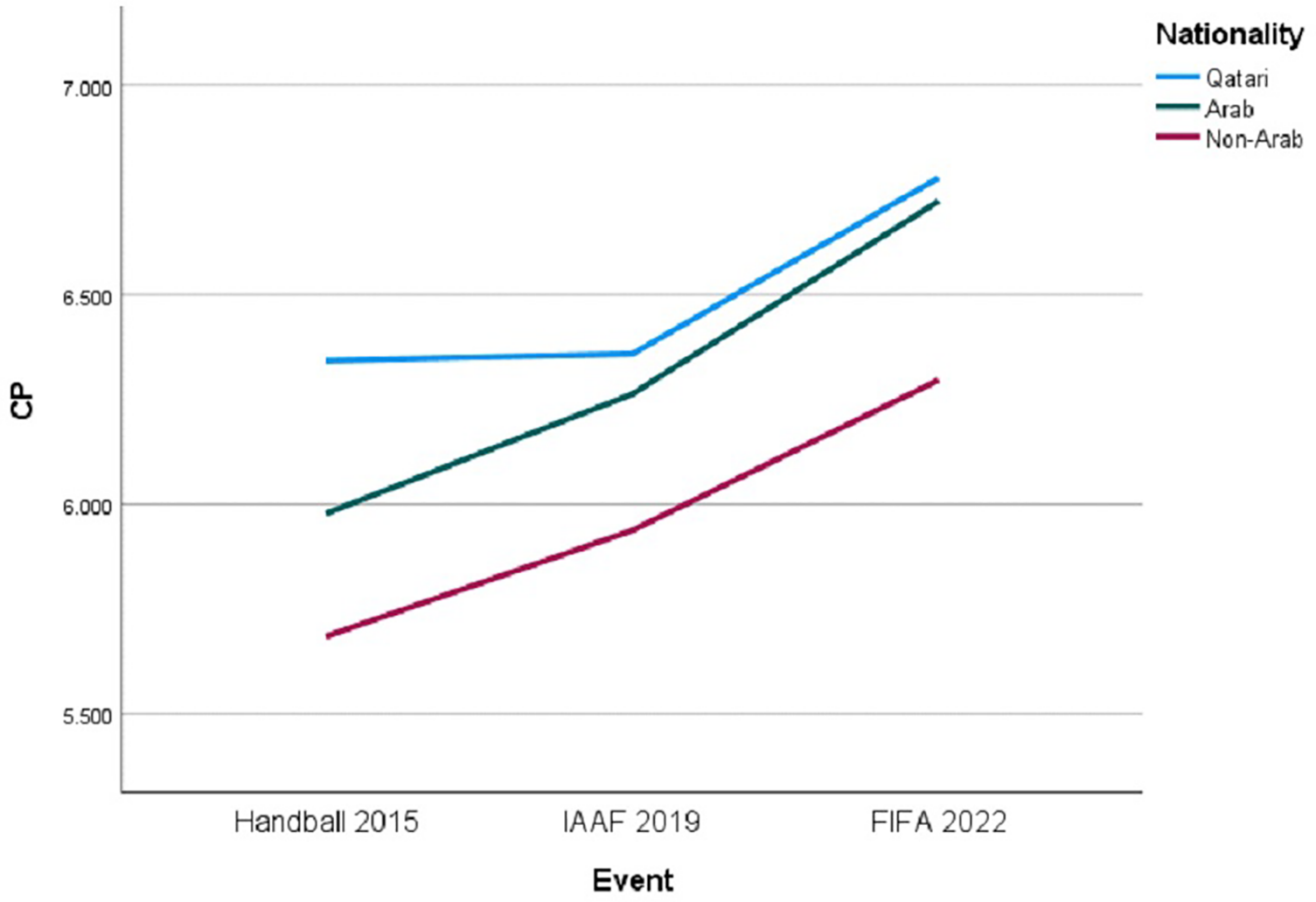
Trends in CP by nationality across major sporting events.

Figure 1 illustrates the trends in CP scores across the different events, segmented by “Nationality.” Despite the overall increase, the interaction between “Event” and “Nationality” did not reach statistical significance, indicating that the growth in CP was consistent across nationalities, regardless of the event [F (1, 755) = 0.709, *p* = 0.586].

Analyzing EC, the corrected model was significant [F (8, 755) = 8.598, *p* < .001], indicating a strong fit see Table 4. The intercept showed a significant average effect [F (1, 755) = 6,501.742, *p* < .001]. The main effect of the “Event” was significant [F (2, 755) = 14.938, *p* < .001], signifying its impact on EC scores. Additionally, the effect of “Nationality” was significant [F (2, 755) = 9.639, *p* < .001], highlighting the distinct impacts of different nationalities on EC scores.

**Table 4:** Analysis of variance for EC by event and nationality.

Source	Type III sum of squares	df	Mean square	F	*p*-value
Corrected Model	142.445	8	17.806	8.598	<.001
Intercept	13,463.987	1	13,463.987	6,501.742	<.001
Event	61.868	2	30.934	14.938	<.001
Nationality	39.919	2	19.960	9.639	<.001
Event * Nationality	19.421	4	4.855	2.345	0.053
Error	1,563.475	755	2.071		
Total	24,053.333	764			
Corrected total	1,705.920	763			

**Figure 2:**
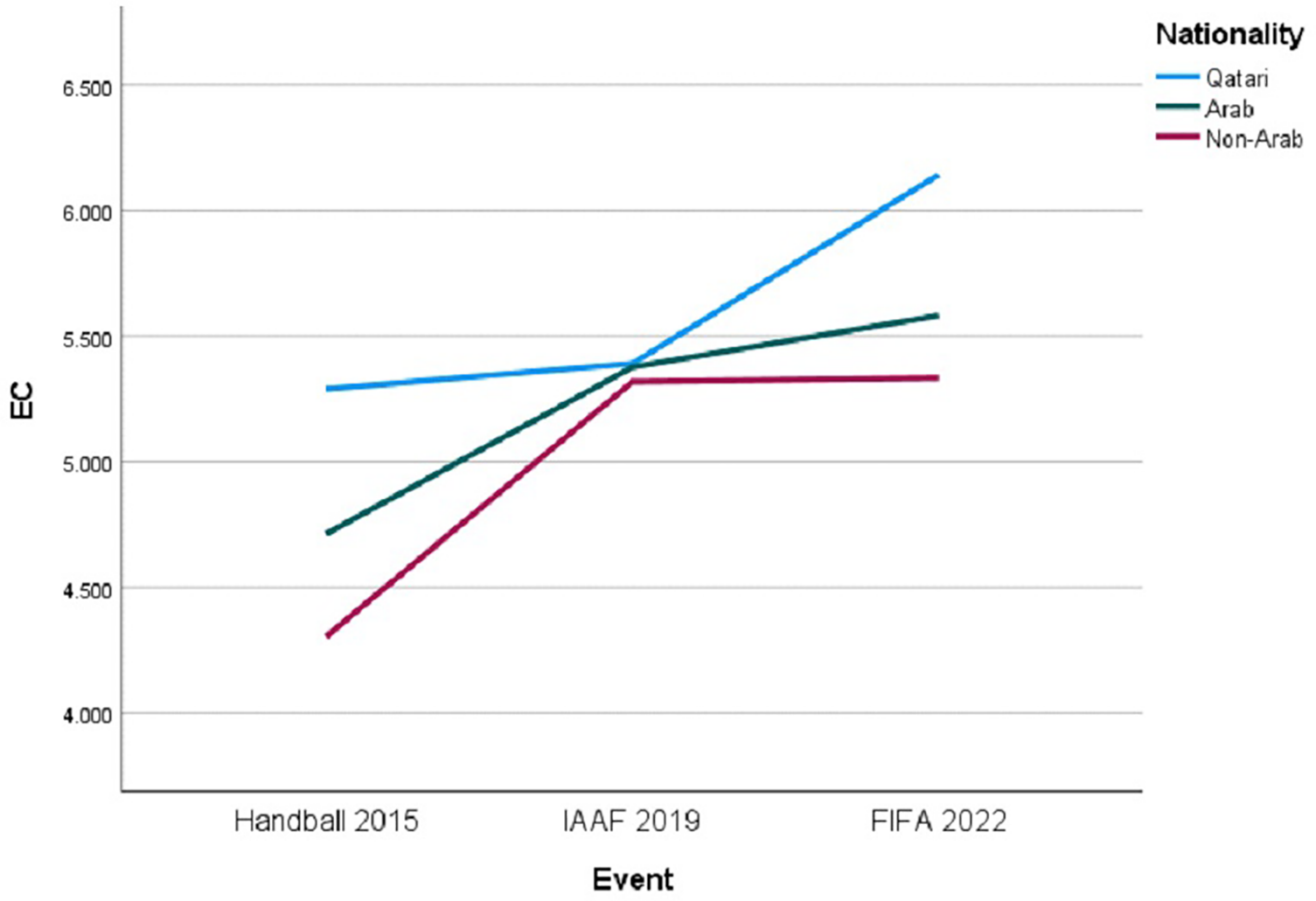
Trends in EC by nationality across major sporting events.

The EC analysis revealed a marginal interaction effect between “Event” and “Nationality” (see Figure 2), approaching significance [F (4, 755) = 2.345, *p* = .053] without meeting conventional statistical thresholds, suggesting a potential trend where the impact of different events on EC scores may not be consistent across nationalities, indicated by varying trajectories for each group.

Table 5 displays the analysis of EE concerning the impact between “Event” and “Nationality.” The corrected model, encompassing main effects and the interaction effect, significantly accounted for variability in EE scores [F (8, 755) = 16.964, *p* < .001], indicating that the model effectively predicts EE.

**Table 5:** Analysis of variance for EE by event and nationality.

Source	Type III sum of squares	df	Mean square	F	*p*-value
Corrected model	334.777	8	41.847	16.964	<.001
Intercept	13,079.759	1	13,079.759	5,302.388	<.001
Event	190.474	2	95.237	38.608	<.001
Nationality	5.267	2	2.634	1.068	0.344
Event * Nationality	39.983	4	9.996	4.052	0.003
Error	1,862.410	755	2.467		
Total	22,822.480	764			
Corrected total	2,197.187	763			

**Figure 3:**
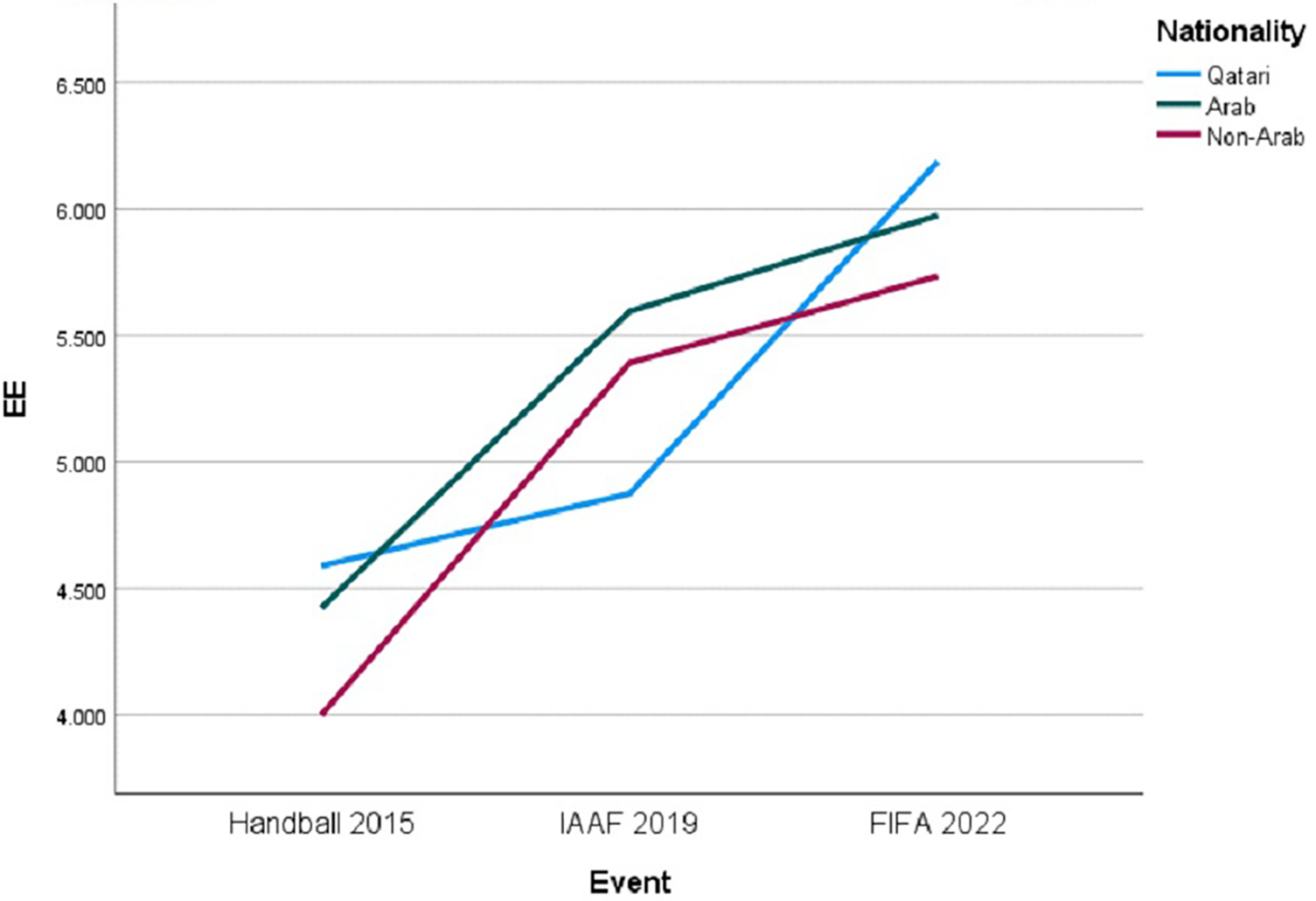
Trends in EE by nationality across major sporting events.

A substantial impact of the “Event” was observed [F (2, 755) = 38.608, *p* < .001], indicating that different types of events significantly influence EE scores. However, the main effect of “Nationality” did not reach statistical significance [F (2, 755) = 1.068, *p* = .344], suggesting that “Nationality” alone did not significantly affect EE scores within this model. The statistical analysis identified a significant interaction effect between “Event” and “Nationality” on EE, as depicted in (Figure 3), with F (4, 755) = 4.052, *p* = .003, suggesting that the events’ influence on EE depends on respondents’ nationalities.

Table 6 displays the variance analysis for PI concerning “Event” and “Nationality.” The corrected model, encompassing main effects and their interaction, significantly predicts PI variance [F (8, 755) = 15.359, *p* < .001], indicating robust predictive power. The highly significant intercept [F (1, 755) = 11,920.173, *p* < .001] denotes a substantial baseline level of PI across all groups. “Event” significantly affects PI [F (2, 755) = 35.880, *p* < .001], and “Nationality” also shows a significant main effect [F (2, 755) = 11.362, *p* < .001], revealing variations in PI scores based on “Nationality.”

**Table 6:** Analysis of variance for PI by event and nationality.

Source	Type III sum of squares	df	Mean square	F	*p*-value
Corrected model	163.073	8	20.384	15.359	<.001
Intercept	15,820.038	1	15,820.038	11,920.173	<.001
Event	95.238	2	47.619	35.880	<.001
Nationality	30.158	2	15.079	11.362	<.001
Event * Nationality	21.899	4	5.475	4.125	0.003
Error	1,002.010	755	1.327		
Total	27,041.889	764			
Corrected total	1,165.082	763			

**Figure 4:**
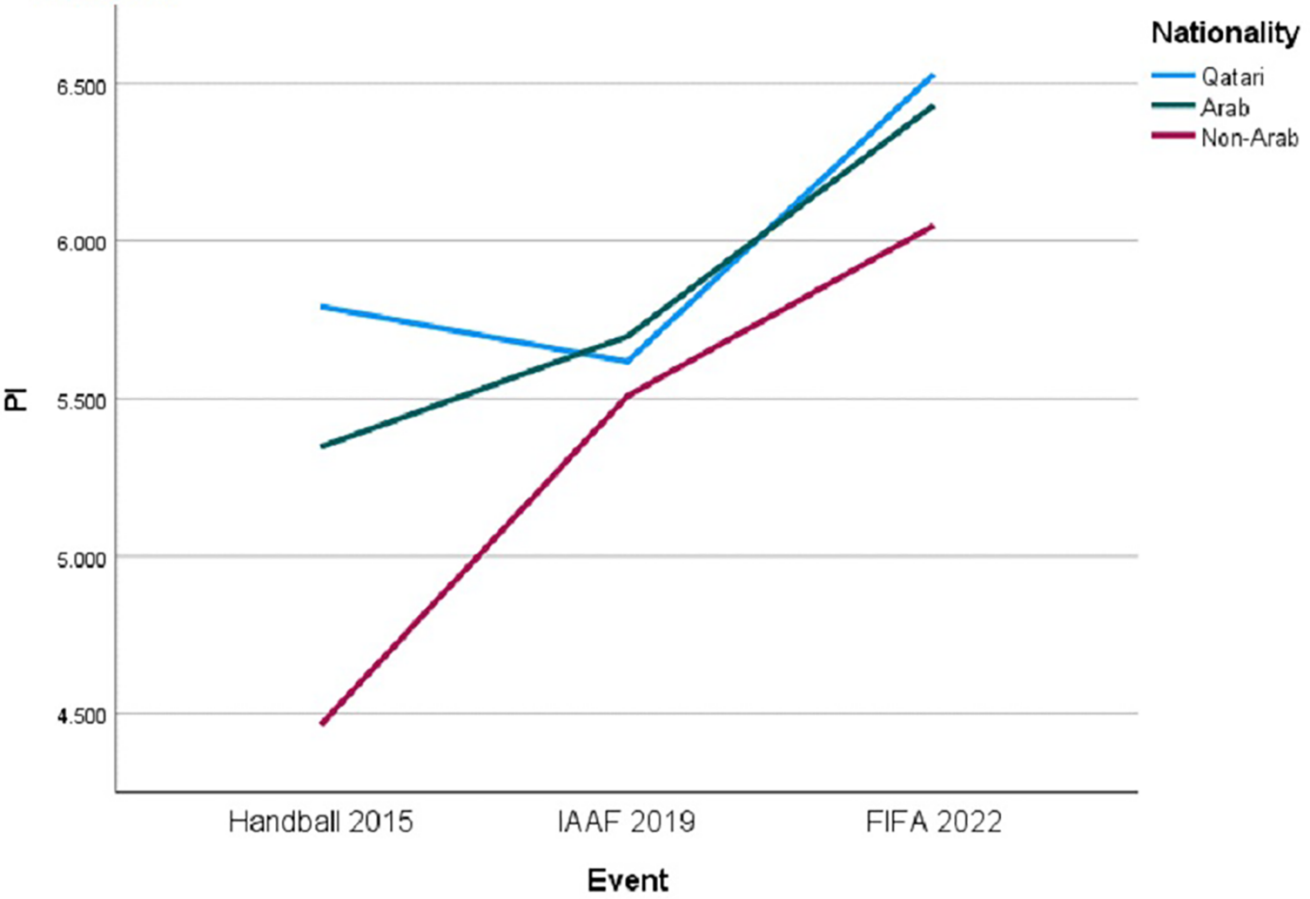
Trends in PI by nationality across major sporting events.

Further investigation into PI uncovered a significant interaction between “Event” and “Nationality” [F (4, 755) = 4.125, *p* = .003]. Figure 4 illustrates distinct PI trajectories, suggesting varied PI aspects experiences for each nationality group.

Table 7 displays CE variance analysis, indicating the corrected model significantly predicts CE [F (8, 755) = 13.853, *p* < .001], explaining a substantial portion of the variance. The intercept is also significant [F (1, 755) = 12,028.783, *p* < .001], denoting a robust baseline level of CE without considering “Event” and “Nationality.” The type of “Event” significantly affects CE [F (2, 755) = 36.254, *p* < .001], showcasing notable impact variations on CE scores. However, the main effect of “Nationality” on CE was not significant [F (2, 755) = 2.488, *p* = .084], indicating no significant differences in CE scores.

**Table 7:** Analysis of variance for CE by event and nationality.

Source	Type III sum of squares	df	Mean square	F	*p*-value
Corrected model	155.920		19.490	13.853	<.001
Intercept	16,923.265	1	16,923.265	12,028.783	<.001
Event	102.012	2	51.006	36.254	<.001
Nationality	7.001	2	3.501	2.488	0.084
Event * Nationality	20.258	4	5.065	3.600	0.006
Error	1,062.208	755	1.407		
Total	28,244.222	764			
Corrected total	1,218.128	763			

**Figure 5:**
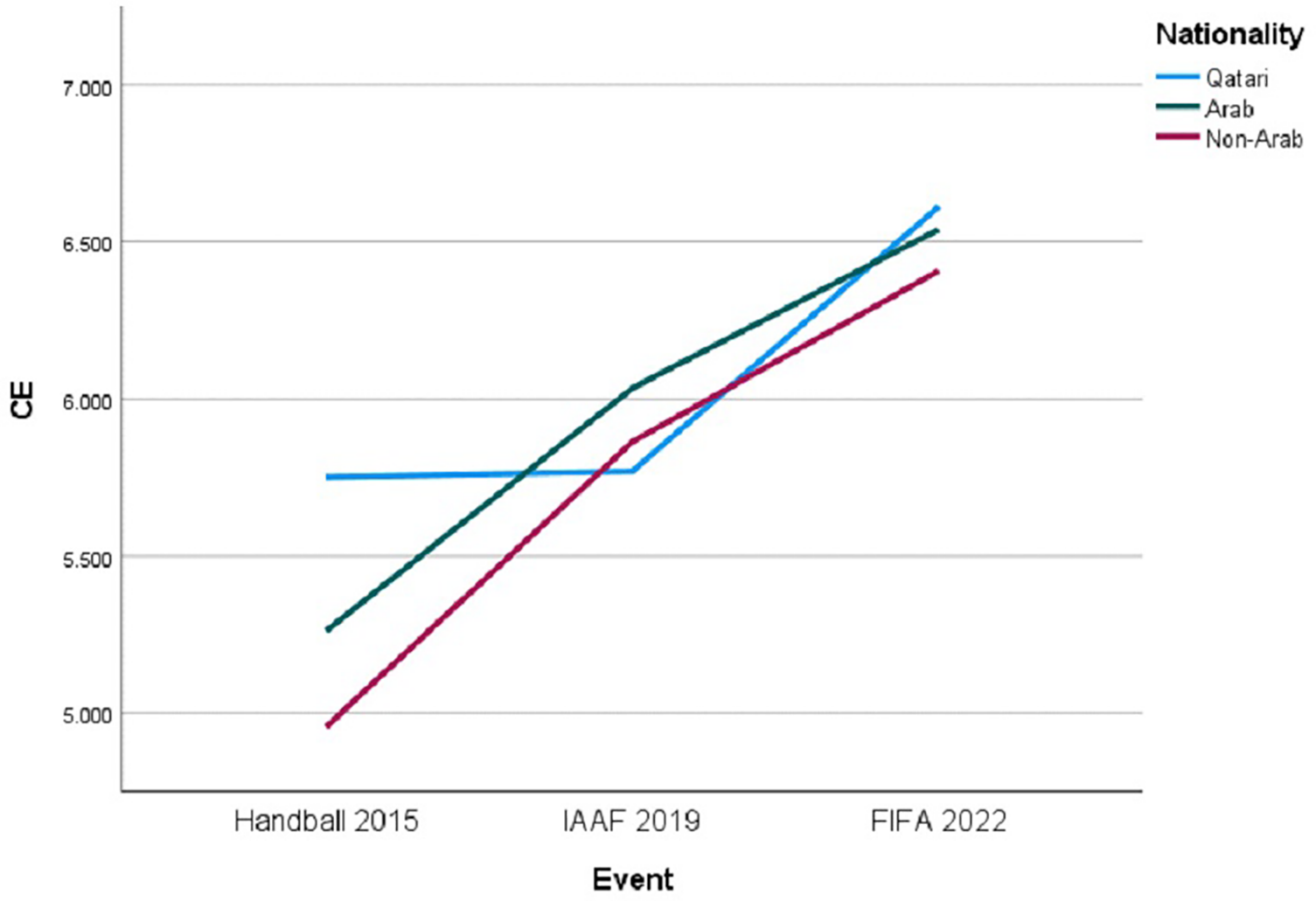
Trends in CE by nationality across three international major sporting events.

The analysis revealed a significant interaction effect between “Event” and “Nationality” on CE, illustrated in Figure 5. The interaction term was statistically significant [F (4, 755) = 3.600, *p* = .006], indicating that participants’ nationalities influenced the level of community excitement elicited by different events.

In assessing the impact of “Event” on the five dimensions, post-hoc analyses (Table 8) revealed a significant increase in CP from HWC 2015 to FIFA 2022 (Mean difference = −0.551, SE = 0.097, *p* < .001). Similar patterns were observed in EC, EE, and CE, indicating heightened excitement in the recent FIFA event. There was no significance between HWC 2015 and IAAF 2019 in PI (Mean difference = −0.113, SE = 0.100, *p* = 0.775). However, significant differences were noted when comparing HWC 2015 to FIFA 2022 (Mean difference = −0.936, SE = 0.109, *p* < .001) and IAAF 2019 to FIFA 2022 (Mean difference = −0.823, SE = 0.102, *p* < .001) in PI.

**Table 8:** *Post-hoc* comparisons of mean differences between events for multiple dependent variables.

Dependent variable	Event (I)	Event (J)	Mean difference (I-J)	SE	*p*-value
CP	Handball 2015	IAAF 2019	−0.123	0.088	0.490
	Handball 2015	FIFA 2022	−0.551	0.097	<.001
	IAAF 2019	FIFA 2022	−0.428	0.090	<.001
EC	Handball 2015	IAAF 2019	−0.381	0.124	0.007
	Handball 2015	FIFA 2022	−0.902	0.136	<.001
	IAAF 2019	FIFA 2022	−0.522	0.127	<.001
EE	Handball 2015	IAAF 2019	−0.650	0.136	<.001
	Handball 2015	FIFA 2022	−1.602	0.149	<.001
	IAAF 2019	FIFA 2022	−0.952	0.139	<.001
PI	Handball 2015	IAAF 2019	−0.113	0.100	0.775
	Handball 2015	FIFA 2022	−0.936	0.109	<.001
	IAAF 2019	FIFA 2022	−0.823	0.102	<.001
CE	Handball 2015	IAAF 2019	−0.342	0.103	0.003
	Handball 2015	FIFA 2022	−1.062	0.112	<.001
	IAAF 2019	FIFA 2022	−0.720	0.105	<.001

To understand the differences among “Nationality” across the 5 dimensions, post-hoc analysis revealed that Qataris reported higher community pride than Non-Arabs (Mean difference = 0.498, SE = 0.116, *p* < .001) within the CP factor. No significant differences were found between Qatari and Arab groups, and between Arab and Non-Arab groups.

In the EC factor, Qataris demonstrated significantly higher scores compared to both Arabs (Mean difference = 0.399, SE = 0.120, *p* = .003) and Non-Arabs (Mean difference = 0.507, SE = 0.163, *p* = .006), while no significant difference was found between Arab and Non-Arab groups. No significant variations were observed in EE and CE factors across all groups.

In the PI factor, a notable mean difference was found between Qatari and Non-Arab groups (Mean difference = 0.526, SE = 0.130, *p* < .001), as well as between Arab and Non-Arab groups (Mean difference = 0.348, SE = 0.143, *p* = .045). However, no significant difference was identified between Qatari and Arab nationals, as highlighted in Table 9.

**Table 9:** *Post-hoc* comparisons of mean differences between youth nationalities for multiple dependent variables.

Dependent variable	Nationality (I)	Nationality (J)	Mean difference (I-J)	SE	*p*-value
CP	Qatari	Arab	0.201	0.085	0.055
	Qatari	Non-Arab	0.498	0.116	<.001
	Arab	Non-Arab	0.297	0.126	0.057
EC	Qatari	Arab	0.399	0.120	0.003
	Qatari	Non-Arab	0.507	0.163	0.006
	Arab	Non-Arab	0.108	0.178	1.000
EE	Qatari	Arab	−0.079	0.130	1.000
	Qatari	Non-Arab	0.028	0.178	1.000
	Arab	Non-Arab	0.108	0.195	1.000
PI	Qatari	Arab	0.177	0.096	0.192
	Qatari	Non-Arab	0.526	0.130	<.001
	Arab	Non-Arab	0.348	0.143	0.045
CE	Qatari	Arab	0.134	0.099	0.527
	Qatari	Non-Arab	0.213	0.134	0.339
	Arab	Non-Arab	0.079	0.147	1.000

## Discussion

Researchers highlight substantial investments in the Arab world, particularly in the GCC, for hosting major sporting events, seen as generating diverse opportunities for development across economic, social, and political spheres (58). Only a few researches have focused on understanding the impact on the community. Ishac et al. (22), and Ishac and Swart (13), assessed the perceived impact on Qatar residents on HWC and IAAF; they found that these events play an important role in enhancing Community Excitement and Pride (50). This study get one step closer by (1) examining perceived impact on Qatar Youth residents through a comparative cross-sectional study, which compares the post-event impact of the HWC 2015, the IAAF 2019, and the FIFA 2022, taking nationality of participants into consideration, and (2) identifying both similarities and differences in youth residents’ perceptions through the different dimensions assessed.

The study’s findings support hypothesis one and show a consistent positive trend in community perceptions across five dimensions, affirming the essence of social sustainability in hosting major sporting events. Kim et al. (59) investigated how mega-events like the F1 Korean Grand Prix 2010 and the 2018 Pyeongchang Winter Olympics influenced residents’ quality of life, finding that community spirit positively affected well-being through enhanced senses of belonging, local attachment, and solidarity, with residents’ recognition of collective and individual community spirit shaping their quality of life. Similarly, after assessing the role of major sport events in regional communities, Wallstam and Kronenberg (53) found that the assessed group perceived a stronger positive emotional impact among different international sport events that took place in the same year.

Furthermore, our study aligns with previous work indicating that hosting major sporting events can increase community pride and attachment over time. These events promote national pride, community cohesion, and resident satisfaction, resulting in increased social capital, community participation, and positive place attachment (39, 40, 50, 51, 60, 61). Such local support for hosting such events provides decision-makers and organizers the necessary validation when bidding to host these international sporting events (50).

Table 2 emphasizes the significant influence of sporting event types on dependent variables, highlighting the impact of both “Event” and “Nationality” on community perceptions. As highlighted by Chun et al. (30) and Wicker and Sotiriadou (31), specific demographic factors such as gender, age, and nationality may influence the expression of higher levels of psychic income. Results shows consistency with Ishac and Swart (13), where the interaction effect underscores varied experiences and perceptions among residents based on specific events and nationalities, indicating a nuanced impact influenced by event significance and residents’ backgrounds. The multivariate effect of “Event” underscores the unique influence of each sporting event on community perceptions. For example, Theodorakis et al. (62) observed heightened football fan engagement among Middle Eastern supporters during FIFA 2022, exemplifying event-specific effects on involvement.

Overall, the data reveals a consistent increase in EC across three periods, indicating that hosting events like the FIFA World Cup likely strengthened residents’ sense of community. Moreover, EE remained consistently high, underscoring ongoing engagement. These findings highlight a sustained excitement and active involvement among residents, possibly influenced by the nature of the events, especially football. Additionally, hosting major sporting events like the FIFA World Cup fosters a sense of community cohesion and solidarity among youth residents. However, it is crucial to recognize residents’ support for tourism relies on the event capacity to uphold and strengthen socio-cultural norms and values. If residents feel that the event is undermining or harming their way of life, it could have negative effects, ultimately harming social sustainability (3, 8, 18). In line with this concern, Al-Emadi et al. (24) highlight a significant issue regarding hosting of the 2022 FIFA World Cup, particularly among Qatar residents in relation to football fan behavior, alcohol consumption and respecting local dress and public behavior. Nevertheless, our study observed a cumulative increase in positive perceptions among youth residing in Qatar, indicating that implemented policies successfully addressed these issues and had a positive impact. This assentation is supported by Ishac et al.'s recent study (63) on public sentiment regarding the 2022 FIFA World Cup among Arabic-speaking Twitter users, primarily from the GCC region. Their findings indicate an initial positive sentiment towards the World Cup among Arabic tweets, with sentiments improving during the tournament and negative sentiments decreasing over time.

Addressing cultural concerns is essential for fostering support and ensuring the success and social sustainability of major sporting events like the FIFA World Cup. Chersulich Tomino et al. (3) found that larger events tend to produce more significant impacts, both positive and negative. Therefore, one might anticipate that the outcomes of the 2022 FIFA World Cup would surpass those of the 2015 HWC and the 2019 IAAF. Yet, it remains crucial to conduct such comparisons when seeking to evaluate their contribution to social sustainability. This comparative analysis is particularly relevant when considering investments in the youth generation.

For instance, Veal et al. (64) found a potential positive link between mega-sporting events and youth involvement in sports, as opposed to adults. Our study findings align with the new strategy outlined by the QOC (2023–2030) which emphasizes continuous encouragement for various segments of society to participate in sports and maintain a healthy life style, and to prioritize the ongoing hosting of major sporting events to stimulate tourism (21).

Examining the nationality of residents can influence how they perceive sports events, as highlighted by McGehee and Andereck (65), who noted that the place where residents grew up influences their perceptions. Assessing evolving impact within societal subgroups in noteworthy; for example, a study followed a semi-longitudinal comparative paradigm between various Olympic games on host city residents found that socially excluded groups rarely benefit from these events (66). This study's results underscores diverse experiences among individuals from distinct national backgrounds, resonating with Ishac and Swart (13) and Ishac et al. (25) emphasizing specific variations among Qatari, Arab, and Non-Arab residents across the dimensions. The outcome aligns with Rasoolimanesh et al. (18) findings of varied perceptions among residents from different sociocultural backgrounds influenced by the place of upbringing (65, 67, 68). The observed distinctions in community perceptions support hypothesis two, affirming that the impact of major sport events on promoting social sustainability and inspiring community development varies significantly based on event types and the nationality of the participants.

By taking into consideration the varied perspectives and experiences within subgroups of residents, decision-makers can make more informed decisions to ensure that the entire society benefits from these events, thereby fostering inclusivity and social cohesion. This approach is in line with Qatar's strategy of investing in hosting major sporting events to meet the specialized demands of the growing sports tourism market. According to Anagnostopoulos and Swart (69), this strategy aims to cater to specialized demands within the expanding sport tourism market. The findings of this study align with the recent government announcement reported by QNA (70) regarding the hosting of the 2023 FIFA Asian Cup and the 21st Asian Games in 2030, along with securing the hosting rights for the 2027 Basketball World Cup, all of which contributes to the long term social sustainability of the country for its residents.

## Conclusion and limitation

This study sheds light on the evolving perceptions of youth residents in Qatar in relation to the social impact associated with hosting major sport events over the past decade. It emphasizes the positive trends in community perceptions, highlighting the role of such events in fostering national pride, social capital, community participation, and positive place attachment, aligning with broader goals of social sustainability and community development (3, 5). The positive trajectory observed in Community Pride and Enhanced Community Attachment suggests active contributions to shaping and reinforcing national identity, encouraging community participation, and fostering positive attachment to the locality (39).

The collective experience of hosting major sport events contributes to a heightened sense of community cohesion and solidarity among youth residents. These findings underscore that residents’ support for tourism is intricately tied to the event's capacity to preserve and strengthen socio-cultural norms and values, thereby enhancing Social Sustainability (3, 8, 18). Similarly, the substantial increase in Pride in Community Infrastructure scores, particularly evident during FIFA 2022, indicates that these events can catalyze notable improvements in public services, sports facilities, and urban regeneration (2), in addition to contributing to the overall promotion and development of tourism and enhancing community infrastructure (71).

These findings underscore the pivotal role of hosting such events in advancing sustainability, highlighting the importance of considering residents’ nationalities. Distinct variations among Qatari nationals, Arabs excluding Qatar nationals, and Non-Arabs underscore the need to further research the residents’ nationality's impact in planning future events for inclusivity and positive outcomes. The study supports Ishac et al. (25) findings on the attitude of the residents of Qatar before FIFA 2022, emphasizing the influence of socio-cultural background on residents’ perceptions. The author suggests continued investment in diverse sport events to engage residents effectively. Additionally, the study advocates for neighboring countries to explore co-hosting major events, leveraging shared socio-cultural backgrounds for regional social sustainability and community development.

To the author’s knowledge, this study marks a significant contribution to research, being the first in the GCC region to conduct a cross-sectional study examining the relationship between various major sports events and residents from different nationalities. The insights gained from this work set the stage for future research, encouraging longitudinal studies to explore further and understand these dynamics.

While this study provides valuable insights into the social impact of sporting events in Qatar, it is essential to acknowledge certain limitations. Firstly, the research primarily focuses on youth residents, and the findings may not fully represent the perspectives of other age groups within the population. Additionally, the study relies on self-reported data, which may be subject to response bias or social desirability bias. The research is also limited to three specific sporting events—HWC 2015, IAAF 2019, and FIFA 2022—and may not capture the diversity of experiences associated with a broader range of events. Therefore, future research might benefit from comparing results among events that have similar size and popularity. This approach would ensure a more equitable and precise comparison. Moreover, the study does not extensively explore the economic and environmental dimensions of the events, which are integral components of sustainability. Future research could delve deeper into these aspects to provide a comprehensive understanding of the overall impact of sporting events on host communities. Lastly, the nature of the study is limited to a decade; an extended time frame could offer insights into the persistence or evolution of community perceptions over a more prolonged period.

Despite these limitations, the findings contribute valuable knowledge for policymakers, event organizers, and researchers interested in the intersection of sports, tourism, and community development. Future studies can build upon this foundation to further explore the multifaceted impacts of sporting events, allowing for the refinement of strategies that promote sustainable practices and enhance positive community outcomes in the realm of sport tourism and sustainability.

## Data Availability

The data supporting the conclusions of this study are available from the corresponding author upon reasonable request.
